# Young women’s social support networks during pregnancy in Soweto, South Africa

**DOI:** 10.4102/phcfm.v16i1.4146

**Published:** 2024-04-29

**Authors:** Khuthala Mabetha, Larske M. Soepnel, Sonja Klingberg, Gugulethu Mabena, Molebogeng Motlhatlhedi, Shane A. Norris, Catherine E. Draper

**Affiliations:** 1 SAMRC/Wits Developmental Pathways for Health Research Unit, Department of Paediatrics, Faculty of Health Sciences, School of Clinical Medicine, University of the Witwatersrand, Johannesburg, South Africa; 2 Julius Global Health, Julius Centre for Health Sciences, Faculty of Health Sciences, University Medical Centre, Utrecht University, Utrecht, The Netherlands; 3 School of Human Development and Health, Faculty of Medicine, University of Southampton, Southampton, United Kingdom

**Keywords:** social support, pregnancy care, wellbeing, maternal health, Soweto, South Africa

## Abstract

**Background:**

Although studies from high-income countries have examined social support during pregnancy, it remains unclear what type of support is received by expectant mothers from low- and middle-income country settings.

**Aim:**

To explore young women’s social support networks during pregnancy in Soweto, South Africa.

**Setting:**

This study was undertaken in an academic hospital based in the Southwestern Townships (Soweto), Johannesburg, in Gauteng province, South Africa.

**Methods:**

An exploratory descriptive qualitative approach was employed. Eighteen (18) young pregnant women were recruited using a purposive sampling approach. In-depth interviews were conducted, and data were analysed using inductive thematic analysis.

**Results:**

Analysis of the data resulted in the development of two superordinate themes namely; (1) relationships during pregnancy and (2) network involvement. Involvement of the various social networks contributed greatly to the young women having a greater sense of potential parental efficacy and increased acceptance of their pregnancies. Pregnant women who receive sufficient social support from immediate networks have increased potential to embrace and give attention to pregnancy-related changes.

**Conclusion:**

Focusing on less-examined characteristics that could enhance pregnant women’s health could help in the reduction of deaths that arise because of pregnancy complications and contribute in globally accelerating increased accessibility to adequate reproductive health.

**Contribution:**

This study’s findings emphasise the necessity for policymakers and healthcare providers to educate the broader community about the importance of partner, family and peer support to minimise risks that may affect pregnancy care and wellbeing of mothers.

## Introduction

Pregnancy is generally viewed as a period that is coupled with various physiological and emotional changes, and these changes can have substantial effects on both maternal and infant health outcomes.^1^ Social support is characterised by the way connections that exist between people who have recurring interactions, fulfil the needs of individuals and this form of support can exist in an emotional, instrumental, affectionate or tangible form.^2^

Social support has been found to have substantial influence psychosocial health of expectant mothers. Expectant mothers have been largely deemed to be increasingly exposed to psychiatric health disorders that are conditions that can be worsened by several characteristics such as monetary and relationship problems as well as poor social support.^3,4^ Some previous research has provided empirical evidence of the link between lack of social support and poor mental health outcomes among expectant mothers. This large body of research has shown that a lack of social support has an impact in the development of depressive symptoms that expectant women experience such as antenatal depression, postpartum depression and subjective wellbeing amongst others.^5,6,7,8,9,10^ Pregnant women who experience a lack of social contact or connection with others, and perceive themselves to have low social support, have increased susceptibility to poor pregnancy outcomes, anxiety, and antenatal depression, particularly if they have a strained relationship with their partner or family, which can subsequently affect various developmental outcomes of their babies.^11,12,13^

Contrary to these findings, other empirical evidence shows that perceived social support safeguards the health and wellbeing of expectant mothers. For instance, social support has been reported to play a crucial role in maternal health and wellbeing,^14^ subsequently resulting in expectant mothers perceiving pregnancy-related changes as less stressful.^15^ In addition, good social support has been found to protect pregnant women from developing psychiatric disorders.^16^ Expectant mothers who receive support from their social networks have an increased likelihood of having optimal or a complete state of mental, emotional and psychological wellbeing in relation to women who lack that support.^17^ In addition, receipt of support in pregnancy can alleviate emotional and physical pressures, subsequently improving the wellbeing of the mother.^18^ The care and support that pregnant woman receive contributes greatly to how they experience their pregnancy.^19^ Thus, social support may result in reduced risks of depression during pregnancy subsequently resulting in positive pregnancy and health outcomes.^20^

Empirical evidence has shown that expectant mothers need support that involves undertaking tangible acts or activities, monitoring, and care which promotes positive wellbeing and health for both mother and child.^21^ In addition, social support is deemed as important in strengthening positive health outcomes in families experiencing events that involve significant changes to their lifestyles such as the birth of a child or rearing of children.^17^

Although there are various studies, particularly from high-income countries, that have examined social support during pregnancy,^20,22,23,24^ previous studies that have been conducted have mostly focused on adolescents or older mothers and have examined social support from a quantitative perspective.^20,25,26,27,28,29,30,31^ There is a dearth of qualitative inquiry that explores young women’s social support networks during pregnancy, through in-depth narrative accounts. Even though young mothers (18–28 years old) are a group less examined, it is important to focus on this group as young mothers are still developing themselves and nurturing a pregnancy and caring for a child can be very challenging when trying to balance their own lifestyles. Young mothers are a developmentally distinct group because although they are better equipped than adolescent mothers in terms of parenting behaviours, they are still not as prepared for optimal parenting as older mothers,^32^ with factors that contribute to this difference remaining largely unexplored. Of note, some studies have focused on the comparison between adolescent mothers and adult mothers by examining various maternity experiences and outcomes in relation to their pregnancies.^33,34,35,36^ Most importantly, young mothers are generally in the emerging adulthood stage, which is a very critical stage in the life course and has the potential to expose them to positive or negative developmental trajectories, in terms of supports, resources, and structures that can or cannot be provided by family, peers, and other broader social services.^37^ Additionally, studies that exist on social support have focused on partners’ role in the health of young mothers which has been limited to paternal involvement defined only through the state of being married or not married or the inclusion of the name of the father on the child’s birth certificate.^38,39^ Moreover, various studies that focus on partner support have not been able to explain partner involvement and pregnancy experiences as well as the type of support that partners offer through more in-depth narrative accounts.^40,41^ In addition, one study that examined perceptions of receipt of social support among a group of pregnant young women who were not in a marital union and were enrolled in a tertiary institution in South Africa, showed that the pregnant students had a perceived need for male partner’s support, with emotional support cited as a major form of support required given that it was likened and associated with receiving affection, adoration and devotion.^42^

The potential role that families play in childbearing practices of young women also merits further research given that many South African young women have been reported not to be married and are also resident in their families’ households during their pregnancy.^43^ Furthermore, several international studies have conducted research on social support initiatives that are specifically run by expectant mothers as well as professional initiatives during pregnancy.^44,45,46^ The findings of these previous studies have shown that expectant women have obtained support from their peers and a therapeutic space in these support groups which has played a significant role in positive pregnancy outcomes and positive emotional wellbeing. In addition, pregnant women have received professional support through individualised education and supportive phone calls and other forms of support which are emotional, affirmational, informational and practical from both mother-to-mother support groups and healthcare providers, resulting in improved Quality of Life (QoL) during pregnancy.^47^ Also, receipt of social support has been mostly evident following the birth of a child.^48^ However, the need for social support is greater in the prenatal period.

While there is extensive literature that on social support initiatives that are received by expectant mothers in high-income countries, it remains unclear what type of support is received by expectant mothers from low- and middle-income country settings, in particular family and peer support among young women. There is limited literature on the social support patterns on the wellbeing of this specific age group and a lack of in-depth understanding of their relationships with peers, families and partners. Given this background, the study’s objective was to explore young women’s social support networks during pregnancy in Soweto.

## Research methods and design

### Study design

A qualitative descriptive design was employed in this study. This study design was employed in this study with the rationale to provide a description of a particular phenomenon under study.^49^ In addition, it is useful in summarising and understanding an area of interest.^50^ Thus, it was relevant to apply in this study given that it aimed to describe and document the phenomenon under study (social support during pregnancy) without providing a broader interpretation of its meaning. Therefore, a qualitative descriptive design was useful in this study as it assisted in the contextualisation of how participants receive social support during pregnancy and provided a picture of how that social support naturally occurs in the participants’ environments. In addition, a descriptive qualitative design affords researchers the opportunity to present the analysis of findings in such a manner that researchers present the data in the raw format to ensure that the data only go through minimal transformation so as not to change the essence of the description of the phenomenon under study.^51,52^

### Study setting

The study took place at the Chris Hani Baragwanath Academic Hospital located in Soweto, Johannesburg, South Africa. It is nested in the Healthy Life Trajectories Initiative (HeLTI), and specifically the *Bukhali* randomised control trial that examines the effects of a complex intervention aimed at optimising the health of young women pre-conception, during pregnancy, and postnatally.^53,54^ Soweto is a historically disadvantaged and underprivileged high-density peri urban area that lies in the outskirts of the city of Johannesburg, with 1.3 million residing in the area.^54,55^ Although Soweto is characterised by diverse economic structures and activities, poverty-related challenges remain rife in the area with food insecurity and unemployment being highly prevalent.^56^ Most of the population residing in Soweto is deemed to have poor or limited access to appropriate healthcare services, particularly youth-friendly services.^57,58^

### Study population and sampling

The study adopted a purposive sampling approach as it assisted in selecting participants who shared similar characteristics and met the selection criteria. The inclusion criteria were young women aged 18–28 years who were enrolled in the trial pre-conception and then fell pregnant within the 18 months of the trial. Eighteen pregnant participants who were in the age group 18–28 years, were recruited from the *Bukhali* trial, from both the intervention and control arms.^53^ This is the age range that was selected as the target group from the initial conceptualisation of the trial. Participants assigned to the intervention arm received health literacy material pertaining to physical and mental health, micronutrient supplements, and free access to HIV and pregnancy testing.^53,54^ The intervention is delivered monthly by community health workers over a period of up to 18 months pre-conception and continuing into pregnancy. Pregnancy-specific material is provided to address healthy diet and healthy behaviours during pregnancy, and antenatal care activities pertain to birth preparation and the child’s arrival.^59,60,61^ Participants in the control arm received information related to life skills and are also offered free HIV and pregnancy testing. Because of the current blinding of the trial, combined results are reported for intervention and control participants.

### Data collection

Participants participated in individual in-depth interviews in the month of August 2021. Questions that guided the interview were generated by C.E.D. (Associate Professor/Reader; PhD), G.M. (Project Coordinator; MA), M.M. (Project Coordinator; BSc). Probing questions were used where needed to enquire closely on various discussion areas. The questions specifically focused on the pregnancy experiences of the participants, their support networks, behaviours pertaining to their health (uptake of antenatal care) as well as where they obtained information on pregnancy. Participants were invited to participate in the interview through approaching them during their in-person monthly sessions or through phone calls. Two local women interviewers (M.M., G.M.) who were familiar with the trial and the local context, conducted the interviews and were able to converse in participants’ home languages in addition to English. Following coronavirus disease 2019 (COVID-19) safety protocols, the participants were interviewed in person in a comfortable and silent room that offered maximum privacy. Interviews were audio-recorded and lasted between 45 and 60 min; notes were taken during each session to document key narratives and participants’ non-verbal cues. A funnel approach to interviewing was used by first connecting with the participants and developing mutual trust, understanding and embracing different perspectives, which enabled participants to speak candidly about their experiences within a safe research environment. Prior to analysis, the recorded narratives of the participants were transcribed verbatim and interviews that were conducted in the participants’ native language were translated into English by a vendor who provided outsourced transcription services.

### Data analysis

The data were analysed through employing an inductive thematic analysis coding approach, with MAXQDA software, version 20.^62^ This is an interpretive method that enables researchers to seek for patterns or meanings from observation or data and then develop explanations from these patterns without relying on a predetermined framework.^63^ The rationale for employing this approach is that its philosophical and methodological foundations are based on learning from experience and the patterns and interrelationships that emerge from these experiences are observed to reach conclusions.^64^

The analysis was conducted in six steps by employing Braun and Clarke’s analysis guidelines.^65^ (1) Firstly, we familiarised ourselves with the data through repeated reading of the transcripts and listening to the audio recordings while simultaneously engaging in notetaking. (2) Secondly, the data were broken down into individual extracts to create codes that were then labelled based on the descriptions of the participants. (3) These codes were then arranged by developing patterns which resulted in the generation of themes. (4) The themes were then evaluated in order to pick out logical and well-organised patterns in the data. This process was also accompanied by combining themes that had the same pattern of meaning by refining, aligning and categorising the themes. Lastly, the themes were determined and identified by matching each participant’s description with the relevant theme. The last step included a write-up which involved providing a concise and coherent narrative of the data.^65^ Initial analysis and coding were conducted by K.M. (Postdoctoral Fellow; PhD) and in the later stages, codes and themes were discussed with the remaining team members; (C.E.D., L.M.S. [Postdoctoral Fellow; PhD], S.K. [Postdoctoral Fellow; PhD] and M.M.) to resolve discrepancies.

Table 1 outlines the themes and subthemes that represent the participants’ perceptions of social support on their pregnancy experiences. They broadly focus on the participants’ relationships during their pregnancies and network involvement.

**TABLE 1:** Themes and subthemes.

Themes	Subthemes
Relationship quality following pregnancy disclosure	Behavioural response of partner following disclosure of pregnancy Behavioural response of family following disclosure of pregnancy
Role and attribute of support networks during pregnancy	Emotional and Instrumental support Informational support (medical and cultural information)

### Ethical considerations

Approval to conduct the study was granted by the Human Research Ethics Committee (Medical) based at the University of the Witwatersrand (M190449). Trial registration: This trial is registered with the Pan African Clinical Trials Registry. The Trial identifier is PACTR201903750173871 and it was registered on the 27th March 2019.

## Results

The sociodemographic factors of the 18 participants have been presented in Table 2. Generally, 22 years was the mean age of the participants, with majority (*n* = 8) of the participants being in the age groups 18–20 years. Most of the participants (*n* = 4) lived with their partners, followed by a few who live with both their mother and siblings (*n* = 3). Majority of the participants were in a relationship but were not living with their partner (*n* = 12). There were no differences in the proportion of participants who reported that the current pregnancy is their first pregnancy (*n* = 9) and those who have given birth before, with the current pregnancy being their second pregnancy (*n* = 9). Most participants were unemployed (*n* = 9), followed by those for whom it was not known whether they were employed or not in any form of employment (*n* = 6), and a few who were in some form of employment (*n* = 3). A slightly larger proportion of participants had a secondary school education (*n* = 10) and only a few (*n* = 2) had a tertiary education.

**TABLE 2:** Participants’ characteristics.

Characteristics	Frequency	Percentage
**Age**		
18–20	8	44.44
21–23	6	33.33
24–26	3	16.67
27–28	1	5.56
Mean age	22	-
**Lives with**		
Partner	4	22.22
Mother and siblings	3	16.67
Mother	2	11.11
Child	2	11.11
Parents and siblings	1	5.55
Mother, aunt and siblings	1	5.55
Grandmother and aunt	1	5.55
Sister	1	5.55
Mother and aunt	1	5.55
Parents and cousins	1	5.55
Grandparents	1	5.55
**Relationship status**		
In a relationship, not living together	12	66.67
In a relationship and living together	4	22.22
Single	2	11.11
**Number of pregnancies**		
1	9	50.00
2	9	50.00
**Employment status**		
Unemployed	9	50.00
Unknown	6	33.33
Employed	3	16.67
**Highest level of education**		
Secondary school education	10	55.55
Some primary education	3	16.67
Primary education	3	16.67
Tertiary education	2	11.11

### Relationship quality following pregnancy disclosure

#### Behavioural response of partner following disclosure of pregnancy

Most participants indicated that their pregnancies were unplanned. Despite this, participants reported feeling happy about their pregnancies. They also described feeling surprised and shocked when they learned about their pregnancy, and their pregnancy experience tended to reflect their overall perception of their relationship dynamics with their close networks. The young women were worried about disclosing the pregnancies to their partners and feared getting negative reactions. However, many of the young women reported that their partners responded with great happiness and joy to the news of their pregnancy and their relationships with their partners remained stable. Thus, the partners’ behavioural response played a key role in making young women perceive that they were supported and safe in their relationships, which subsequently fostered feelings of happiness and acceptance of the pregnancy:

‘Things are fine between me and my partner, normal as before. Since he accepted, he feels like he is okay, because he is also very supportive’ (Participant 1, age 22 years, secondary school qualification, employment status unknown).

‘He is also excited because it’s what he’s been hoping for and to him it’s the first child. We’re all okay, and, we stuck together, he’s supportive. We stay together, we do all things together, we’re not apart; only if he’s at work. Like we’re all on the same page.’ (Participant 2, age 25 years, secondary school qualification and unemployed)

However, contrary to these findings, other participants indicated that their relationships with their partners changed following the disclosure of their pregnancy. Partners distanced themselves from the young women resulting in a dissolution of the relationship. Such circumstances resulted in the young women having negative pregnancy experiences and not accepting their pregnancies:

‘My relationship with my partner was fine, until I was pregnant. I told him about the pregnancy, and he did not want to be involved, and I just cancelled him out of my life.’ (Participant 3, age18 years, secondary school qualification, unemployed)

‘I told the child’s father that I am pregnant then he decided that we should stop seeing each other. He said to me in March, he does not want this child, he won’t be able to help me so it’s best that I get an abortion, so I asked him that when I get an abortion, am I supposed to do it alone, because after that you have to get me cleansed, and then he said to me that I will find my own way, he is not interested, I must just leave him alone.’ (Participant 4, age 20 years, primary school qualification, employment status unknown)

#### Behavioural response of family following disclosure of pregnancy

Some participants reported that their families were shocked at the news of the pregnancy while some reported that their families were highly disappointed with them as they had wished that the young women could have focused on advancing their education or career. Despite these circumstances, the relationships between the young women and their family members remained stable and most were resident in the same households as their families. The participants had the perception that they were supported by their families and perceived a sense of closeness which contributed positively to their pregnancy experience. Most participants could confide and seek guidance from their families, and this played a significant role in their pregnancy experience.

‘I thought that my mother was going to be angry or chase me away or something, but then she sat down and spoke to me and told me to keep it, because she has never made anyone do it, she is the first born so why does she have to judge me, life will go on and we will be alright.’ (Participant 4, age 20 years, primary school qualification, employment status unknown)

‘My mother asked me that she heard my grandmother telling her that I am pregnant, and I said yes, I am pregnant. She said okay, there is nothing I can do since you are already pregnant. I will not tell you to abort, when you are pregnant, you are pregnant. A baby doesn’t have to be aborted and then she said that they will support me where they are able to support me, and that I will understand, and I told her that I don’t have a problem if you are going to support me, which is the only thing that I need, and that was it.’ (Participant 8, age 23 years, secondary school qualification, unemployed)

‘My cousins said whatever decision I take they will support me, whether I keep the baby or not’ (Participant 16, age19 years, secondary school qualification, employment status unknown).

Contrary to these findings, the narrative of one participant showed that her relationship with her family changed drastically following disclosure of her pregnancy, resulting in a dismantled relationship and poor communication, and the participant having a bad pregnancy experience:

‘I had a bad pregnancy. My mom she was disappointed, very disappointed I won’t lie but not as much as my dad, my dad yoh he was really like shattered, he was like yoh in so much disbelief, he didn’t believe. It was hectic, hectic to a point where my dad cut me off financially, uh nobody spoke to me until now that I’m seven … like last month when I was 7 months; so, nobody spoke to me basically for like 4 or 5 or 6 … ja 3 months. No support at all.’ (Participant 17, age 20 years, secondary school qualification, employment status unknown)

### Role and attribute of support networks during pregnancy

#### Emotional and instrumental support

Participants perceived that both their partners and families are a key pillar in supporting the participants during their pregnancy and helping them navigate through challenges associated with pregnancy. Although the participants received financial support from their families and partners, participants mostly perceived receiving emotional support which contributed significantly to their pregnancy experience. Some participants perceived receiving most support from their partners, some from their families and some perceived having an equal balance of support from both networks. In addition, partner and family engagements included provision of funds to meet healthcare needs, food security and provision of a conducive living environment. These narratives suggest that partners and families demonstrated an increased understanding and concern for the young women during their pregnancies, which contributed immensely to positive pregnancy-related changes, being happy, and accepting the pregnancy.

‘The support I get from my mother, it is better. Makes everything easier. She cooks for me and makes sure I eat healthy’ (Participant 10, age 23 years, secondary school qualification, unemployed).

‘He supports me, he’s there emotionally, physically, financially he supports me with everything that I need, he is there, we talk, his there, he is open, yeah. I am also under his medical aid.’ (Participant 6, age 22 years, primary school qualification, employed)

‘He is just strong at being a very supportive partner. In terms of my education and job hunting and he has been watching my diet since pregnancy. He is my dietician on that one, emotional support he is there.’ (Participant 12, age 22 years, tertiary education, employed)

Although Participant 12’s partner plays a key role in providing her with support in terms of her furthering her education, looking for employment and ensuring that she participates in healthy eating behaviours and providing for her financially, the participant perceives that her partner is not emotionally invested in the ‘physical pregnancy’:

‘He doesn’t want to have those intimate experiences or moments with the baby. Because sometimes okay, the first time I experienced the baby kicking, I asked him to come and feel and he was just okay. He doesn’t ask much so I decided I’m no longer inviting him. He does ask on when last I felt the baby kicking and I would tell him that I felt it earlier on. He would then say I didn’t tell him but to me it would make no difference because he just touches my stomach and say okay!’ (Participant 12, age 22 years, tertiary education, employed)

In addition to receiving both instrumental and emotional care from their family members and partners, two participants also indicated receiving support from the families of their partners. The participants perceived that their partners’ families showed concern, acceptance, affection and warmth to them and also provided them with emotional support which suggests that the partners’ families felt a sense of responsibility and obligation towards the unborn child:

‘My partner, my family and partner’s family support me emotionally. When I have something that bothers me from home or with my friends, I speak to him, and he can support me. Makes me feel happy cause it is rare to find someone who is there with you and support you, especially during tough times. Yes, I can talk to them.’ (Participant 1, age 22 years, secondary school qualification, employment status unknown)

‘I think they are giving me love and they are trying to make me feel comfortable with the pregnancy, because at first I was like no, my age and a second child, I won’t be able to, and then my mom, his mom, they are there for me, that it’s something that happens, that you want to study further, it’s not something that will stop, like they give me, they encourage me you see, we are there, we will stay with the child, you can carry on with what you want to do and whatnot and whatnot, you see, so yeah.’ (Participant 10, age 23 years, secondary school qualification, unemployed)

Despite these two narratives, one participant perceived that she has never had a meaningful connection with the father in either of her two pregnancies, nor received any form of social support from him, his family, or her own family. Such adversities have affected her resulting in her feeling less happy about her pregnancy:

‘I’ve been having fights with the baby’s father, so I have lost interest in the pregnancy. Another thing is the lack of support from his family and mine. I sometimes feel depressed like sad; I lost hope and I even regret why I fell pregnant.’ (Participant 11, age 25 years, secondary school qualification, unemployed)

#### Informational support

Most of the participants perceived receiving peer support through friends who provided support to the participants through sharing knowledge based on their own previous experiences, emotional support, social interaction or practical help, and information on pregnancy care and pregnancy education from trial staff (details about intervention and control arms removed from quotes to protect blinding). In addition, the participants indicated that the informational support they received from the trial and health services has contributed greatly to them having positive feelings about their pregnancies, feeling less isolated, and having improved emotional wellbeing:

‘I got most of the information from [*the trial*]. That is the one that made me understand most about the baby. That when the baby gets to certain months, they can listen to sounds, they can bond with you, the hair grows.’ (Participant 3, age18 years, secondary school qualification, unemployed)

‘The information that I have is about the growth of the baby, the change of the woman’s baby, I got it from the clinic, obviously the card that I have, and then how the belly grows, I have got a book from [*the trial*], we went through a lot of pages, it shows which month the baby grows like this.’ (Participant 6, age 22 years, primary school qualification, employed)

‘I got support from [*the trial*] and the clinic. On how to raise the child and having to breastfeed the baby up until six months for them to be healthy. Like how I am supposed to eat, I must avoid stress most of the time.’ (Participant 14, age 23 years, secondary school qualification, employed)

Other participants reported that they received information on pregnancy care from their friends. These narratives showed that the emotional connection that they have formed with friends who share similar experiences of pregnancy has contributed greatly to positive pregnancy experiences:

‘My friends fell pregnant when we were still at school, and so we talk about everything. They are also open. I have open friends. So, if we have challenges, we talk and help each other. So, most of the information I get from them which has contributed positively to my pregnancy.’ (Participant 1, age 22 years, secondary school qualification, employment status unknown)

‘Some of the information I got it from my other friend. She has a baby, so she has the experience’ (Participant 18, age 28 years, secondary school qualification, unemployed)

Some participants also perceived receiving informational support from their families around pregnancy, through families imparting information on various traditional or cultural pregnancy practices that the participants had to follow to have a positive pregnancy experience. The women’s narratives around this showed that the cultural knowledge they possess around pregnancy has markedly influenced their pregnancy experiences. It has resulted in young women accepting their pregnancies and protecting their pregnancies from a spiritual perspective:

‘The cultural practices that we follow at home and in my church during pregnancy is that they tie you with a band on your waist to protect you, you drink water which they prayed over, in case you come across evil and being pregnant, the baby catches a lot of things.’ (Participant 1, age 22 years, secondary school qualification, employment status unknown)

‘My mother gave me remedies to drink and waist braces. She said they protect the child from evil spirits’ (Participant 9, age 20 years, some primary school education, unemployed).

‘My family took me to “someone” [*traditional healer*] just so they can “strengthen my pregnancy.” He said I must come with milk and eggs, ja and then he mixed them, and he said I must bathe with them, and then he said I must also bring Vaseline as well, and then he mixed it, I can’t remember what herbs he used, but then told me to lather my body with it every night.’ (Participant 13, age 21 years, some primary school education, employment status unknown)

‘There is a man that my mom took me to. He gave me water to drink. Water mixed with some soil and a string. It was to tie the pregnancy because they suspected that I might miscarry this one as well.’ (Participant 14, age 23 years, secondary school qualification, employed)

‘My elders do tell me that I don’t have to show my stomach off all the time because people out there can prevent me from delivering the baby and all that stuff; ja and that I should wash myself with *isiwasho* [*traditional medicine infused water*]. It’s to protect the baby they say, ja and protect delivery.’ (Participant 16, age19 years, secondary school qualification, employment status unknown)

One participant reported that she received no informational support from peers, health services, members of her family, or from her friends, but perceived gathering the information herself through her own learned experiences during her first pregnancy:

‘For me it happened naturally. It was just general knowledge, having to understand things the way they should be. With my firstborn, I was breastfeeding him and I learned how to take care of him.’ (Participant 11, age 25 years, secondary school qualification, unemployed)

## Discussion

The aim of this study was to explore pregnant young women’s social support networks during pregnancy in Soweto, South Africa. Our findings showed that pregnant young women’s perceived support from members of various relationship networks contributed greatly to their pregnancy experiences, with receipt of social support leading to increased acceptance of pregnancy and a lack of social support resulting in negative pregnancy experiences. This finding is in line with existing empirical evidence which demonstrates that social support plays a major role in influencing women’s experiences during pregnancy, with women who receive sufficient support portraying increased potential to embrace their pregnancy and those who lack support being exposed to adverse maternal health outcomes including use of illicit substances, increased exposure to poor mental health outcomes, and adverse birth outcomes.^18,28^ This current study further found that other young women received emotional and instrumental support from both their partners and families while other participants perceived receiving support only from their partners or only from their families. The support received contributed greatly to increased acceptance of the young women’s pregnancies with most of the young women portraying potential for increased parental efficacy.

This finding is strongly congruent to a study that examined parent efficacy and social support systems of young mothers which found that support is made available to young mothers although the nature and type of support received somewhat varies.^66^ A possible explanation for this increased acceptance and potential parental efficacy particularly among young women who received support from their partners can be attributed to the fact that partner support, stability and dependability play a key role in positive pregnancy experiences and acceptance of pregnancy.^67^ Moreover, an engaged partner who cares about the process of pregnancy contributes positively to an expectant woman, which subsequently results in positive pregnancy experiences.^67^ Thus, it can be suggested that young expectant women value the attachment that they have with their partners as they largely deem these supportive networks as playing a key role in helping them to cope better with their pregnancies. The findings of this study thus address a critical gap in the literature as there is a dearth of literature of the nature and receipt of social support from a qualitative perspective, particularly among the population group of pregnant young women in South Africa.

Our study findings further showed that male partners were cited as the most important sources of social support with the type of support received being of an emotional and instrumental nature. This finding is in line with a previous study that was conducted among pregnant young women attending a tertiary institution in South Africa which found that psychological support received from male partners is largely deemed as a major form of support required given that it was likened and associated with receiving affection, adoration and devotion.^42^ Though the study’s finding is supported by previous literature, it is largely contradictory to what has been observed in previous literature, which indicates that unmarried fathers have increased odds of being less involved during pregnancy than fathers who are in a marital union, thus having no obligation towards providing social support to the mother during pregnancy.^68^ Our findings around partner support in this study are highly unanticipated as South Africa is largely documented to have high rates of father absenteeism, when compared to other countries^69^ which can be partly traced back to the apartheid regime, urbanisation and labour migration, which had a strong impact on family life and composition and changed the manner in which families functioned, with these changes being more prominent in the roles of fathers, mothers and extended family members.^70^

This lack of participation in parenthood stems from the fact that most young fathers never knew their fathers which has contributed greatly to their lack of guidance and experience on father roles and responsibilities.^70^ Other constructs of fatherhood including masculinity constructs such as aggressiveness, emotional detachment, dominant and tough behaviour as well as a lack of affectionate communication^71^ deem male emotional and nurturing forms of support as ‘soft’ behaviors^72^ which suggests that fathers must be emotionally distant.^73^ This subsequently creates an obstacle in the fulfilment of positive father–child relationships. In addition, non-involvement of young fathers can be attributed to the increase in poorly paid, unprotected and insecure jobs and enduring rates of impoverishment and inequity in South Africa that place men at a disadvantage of being able to provide adequate care to their families.^74^

Contrary to these findings, other young women reported a lack of emotional and instrumental support from their families as well as strained relationships with their families, following the disclosure of their pregnancies. This finding can be explained by previous literature which has shown that childbearing among young women is largely associated with social stigma and familial shame.^75^ Moreover, from a socioeconomic standpoint, childbearing among young women has been found to have unfavourable results on the child, the young mother, and extended family members.^76^ This is because the family ends up having to assume the responsibility of shouldering the financial burden of childrearing.^76^ Therefore, the findings of this study suggest that families punish young girls who fall pregnant by giving them poor or no support which leads to challenges for young mothers to accept their pregnancies.

Overall, our findings suggest that experiences of receipt of social support among young women differ greatly, with the type and source of social support not being universal. It is thus pivotal to consider the significance of each perceived source of support individually because pregnant young women are not a homogenous group. They have a variety of demographic and familial characteristics, different relations with their families and partners, and the experiences and context in which they live may differ which contributes greatly to positive pregnancy experiences or lack thereof.

As experiences of pregnancy are vastly different and complexities around family and partner dynamics create different realities for these young women, social support needs to be relevant to the different realities of young women. Focusing attention on pregnant women to identify those who experience poor levels of social support along with the provision of community-based support services in collaboration with partners and families, may help foster positive pregnancy experiences with pregnant women taking an initiative to focus on caring for their pregnancy. Furthermore, interventions that urge young women to engage with their personal networks are needed. Health professionals can engage directly with partners and families of young women, by providing information and training on how to support the young women whilst being sensitive to the beliefs and cultural values of these sources of support.

In sub-Saharan Africa, sociocultural practices often promote taking on positive health behaviours which serve as a protective mechanism for expectant mothers’ health-related decision-making. For instance, grandmothers are the most important social networks in sub-Saharan Africa. They make a substantial contribution as advisers and caregivers to young women who greatly influence maternal health-related practices specifically regarding pregnancy.^77^

Thus, the cultural contexts that inform the practices imparted to expectant mothers to promote positive pregnancy outcomes and experiences can be leveraged by introducing intercultural approaches or culturally appropriate models that incorporate cultural knowledge and responsiveness (i.e. how the various cultural practice has markedly influenced the pregnancy outcome or experience) into health education. This could be done through engaging in joint collaborations with programmes that focus on the health and wellbeing of mothers that serve settings in South Africa that have poor or no resources, including this study’s setting (Soweto) to make modifications or adjust interventions that address the feelings, attitudes and beliefs of people within the relevant target populations. This approach would channel innate shared values and resources which would play a pivotal role in influencing expectant young mother’s decision-making concerning health-seeking behaviours and would increase the need for health services, as health-seeking behaviour is not only limited to individuals but lies within socio-cultural contexts.

Thus, receipt of social support has the potential to build resilience among the young women, both at an individual and community level, which would enhance their ability to manage adversity. Therefore, exposure to supportive social networks could ameliorate the threats associated with these social challenges as receipt of social support from various social networks could potentially help strengthen the behavioural and functional aspects of these young women’s relationships with their respective social networks through creation of clear and accessible social ties. Health workers should also be encouraged to mobilise the broader communities of these young women through mass-media campaigns and discourses that promote the need for the supportive role of partners and families and empower young women to have agency and autonomy over the decisions they make on their reproductive health. Male engagement during pregnancy could be promoted by capacitating men with the skills that facilitate and support improved self-care of women during pregnancy, improve home care practices of the young women, understanding of the importance of seeking healthcare and the stages of pregnancy as well as how to foster positive connections with the young women, most importantly development of stronger couple relations. These initiatives would show that programmes are not only highly efficient but respond to the needs of the local communities.

The strength of this study is an exploration of young women’s social support networks during pregnancy, as it helped the researchers to gain a nuanced understanding of participants’ views around the phenomenon under study. This provides insight into a particular context, although transferability to other contexts should not be assumed, particularly as participants were part of a trial. The study’s limitation was that the support networks of the young women were not included as study participants. These individuals could also provide their perspectives on how they believe their provision of social support has had a major influence in the pregnancy experience of the young women.

## Conclusion

The existence of social support has a major influence on pregnancy experiences. Pregnant women who receive sufficient social support from immediate networks have increased potential to embrace and give attention to pregnancy-related changes. This will, in turn, foster positive behavioural outcomes that encourage engaging in good pregnancy care practices and acceptance of motherhood. The important role of maternal support during pregnancy suggests that the wider community needs to be educated by policymakers and healthcare providers about the importance of partner, family and peer support to minimise risks that may affect pregnancy. Future research should be conducted that explores the dynamics of social support within various family structures. Improving receipt of social support by young mothers could also enhance the promotion of physical and mental health of mothers which would subsequently result in the engagement of healthy behaviours in the perinatal period and positive birth outcomes.
